# MSLNet and Perceptual Grouping for Guidewire Segmentation and Localization

**DOI:** 10.3390/s25206426

**Published:** 2025-10-17

**Authors:** Adrian Barbu

**Affiliations:** Statistics Department, Florida State University, Tallahassee, FL 32306, USA; abarbu@fsu.edu

**Keywords:** fluoroscopy, guidewire segmentation, guidewire localization

## Abstract

Fluoroscopy (real-time X-ray) images are used for monitoring minimally invasive coronary angioplasty operations such as stent placement. During these operations, a thin wire called a guidewire is used to guide different tools, such as a stent or a balloon, in order to repair the vessels. However, fluoroscopy images are noisy, and the guidewire is very thin, practically invisible in many places, making its localization very difficult. Guidewire segmentation is the task of finding the guidewire pixels, while guidewire localization is the higher-level task aimed at finding a parameterized curve describing the guidewire points. This paper presents a method for guidewire localization that starts from a guidewire segmentation, from which it extracts a number of initial curves as pixel chains and uses a novel perceptual grouping method to merge these initial curves into a small number of curves. The paper also introduces a novel guidewire segmentation method that uses a residual network (ResNet) as a feature extractor and predicts a coarse segmentation that is refined only in promising locations to a fine segmentation. Experiments on two challenging datasets, one with 871 frames and one with 23,449 frames, show that the method obtains results competitive with existing segmentation methods such as Res-UNet and nnU-Net, while having no skip connections and a faster inference time.

## 1. Introduction

Heart disease is still the number one cause of death in the US, accounting for 22% of the deaths in 2023 [1]. Heart disease is caused in main part by atherosclerosis, in which cholesterol deposits line the heart arteries, in time occluding them and starving the heart of oxygen. Currently, there exists no medication to reverse atherosclerosis, only medications to prevent heart attacks and strokes.

The occluded arteries can be repaired either via bypass surgery, an invasive procedure that stops the heart and bypasses the clogged arteries, or by coronary angioplasty, a minimally invasive procedure that places stents (little specialized springs) at the occluded locations to keep the arteries open. The coronary angioplasty procedure is performed through catheters inserted in the body through an artery, and is monitored using real-time X-ray called fluoroscopy.

Inside the catheter is a thin wire called the guidewire that is used to penetrate the occluded location and guide different tools, such as a stent or a balloon, to perform the procedure. Because X-rays are harmful in high doses, the energy and duration of the X-ray are kept to a minimum, which results in noisy images and decreased guidewire visibility. For these reasons, fluoroscopy can be considered a noisy imaging sensor.

Finding the guidewire automatically is important for different purposes, such as image augmentation, 2D-3D integration, etc. However, there are different levels of finding the guidewire.

The lowest level is guidewire segmentation, where just the pixels of the guidewire are desired to be found. A higher-level task is guidewire localization, where a parametrization of the guidewire is desired to be found, either as a spline or another parametrized curve representation. This task is especially challenging because there might be multiple guidewires present in the image, and a separate curve is required for each of them. At the same time, because of the noisy nature of the fluoroscopy images, large parts of the guidewire might be invisible, and seemingly disparate guidewire segments need to be combined into the same curve based on good continuation.

Finding a parameterization of the guidewire is important for certain tasks, such as guidewire tracking or 2D/3D registration.

For this purpose, the paper brings the following contributions:

1. It introduces a guidewire segmentation method that uses two prediction outputs from a residual network or other feature extractor: one that predicts a coarse segmentation directly from the encoder output, and one that refines the coarse segmentation only at the relevant places using a single convolutional layer. In contrast to the UNet or other segmentation methods, this architecture does not have any skip connections, making it simpler and faster to train.
2. It introduces a method for guidewire localization based on perceptual grouping of the curves extracted from the guidewire segmentation output. The novel perceptual grouping method uses a continuity measure to score what curves might belong to the same guidewire and the Hungarian algorithm to match the curve ends for grouping. This way, the proposed perceptual grouping method uses the Hungarian algorithm to find the global minimum of a cost function, which is a more principled approach than the heuristic-based methods that do not minimize a cost function, or the greedy methods that usually cannot find a global minimum.
3. It performs experiments on two datasets, showing that the proposed segmentation usually outperforms other existing segmentation methods, including the Res-UNet [2], a UNet with residual layers, and the nnU-Net [3], a well-celebrated segmentation method that is the state of the art for many medical imaging segmentation problems.
4. It also performs localization experiments and extensive ablations on the same datasets, showing that the proposed perceptual grouping obtains competitive localization results with a small average number of curves per image.

The proposed segmentation and localization methods are presented as fundamental research aimed at advancing the knowledge of how to find guidewires, catheters, or other curves in images. More research and evaluation are needed to introduce these methods into clinical practice.

## 2. Related Work

While there are quite a lot of works in guidewire segmentation, we are only aware of a small number of works on guidewire localization.

### 2.1. Guidewire Segmentation

The UNet [4] is a U-shape CNN architecture that has an encoder-decoder structure. The encoder has convolutional blocks followed by max-pooling to gradually reduce the spatial resolution of the output while increasing the number of channels. The decoder has a mirror architecture with the encoder, with the same number of blocks, and uses skip connections to bring information from the corresponding encoder layer, which are combined with the upscaled inputs from the previous block using convolutions.

The Res-Unet [2] is a modified UNet that was introduced for catheter segmentation. As opposed to the standard UNet that uses convolutions for the encoder and decoder blocks [2], uses residual blocks [5], which are convolutional layers that sum the input to the block output for improved back-propagation.

A simple method based on image processing was introduced in [6] for guidewire segmentation and localization. The segmentation is obtained by applying a Frangi filter [7] and using a *k*-nearest neighbor classifier to classify 20 × 40 pixel patches centered at high response locations and oriented by the Frangi filter orientation. The method is evaluated only on 8 image sequences and successfully detects the guidewire in 83.4% of the frames.

A steerable CNN was introduced in [8] as the first level of screening for guidewire segmentation, where the CNN’s filters were steered to align with the guidewire direction for better accuracy. The paper used 25 × 25 pixel patches for predicting whether the center pixel is on the guidewire or not, and was focused on obtaining a good precision for 90% recall. In contrast, the proposed segmentation method uses a fully convolutional ResNet that takes a much larger context into account and is able to obtain much better guidewire segmentation results that trade off precision with recall.

A version of UNet was used in [9] for catheter segmentation. The method used a small UNet and transfer learning from synthetic data or phantom data, to obtain results similar to [2] on a catheter dataset.

In [10], the authors propose a two-phase guidewire segmentation method that uses a neural network to predict a binary indicator whether overlapping 32 × 32 patches contain the guidewire or not, and a UNet to obtain the segmentation result on the patches that are predicted positive. The paper does not specify how to combine the obtained overlapping segmentations, and also misses details on data augmentation during training, and has no code available. In contrast, the guidewire segmentation part of our work uses a single neural network to predict, at the same time, a binary indicator on non-overlapping patches and to obtain the final segmentation on the predicted positive patches, without any UNet-like decoder and without any skip connections. Moreover, our architecture is fully convolutional and does not need to extract image patches, gathering larger context and obtaining much better segmentation results, besides being more computationally efficient.

Another UNet architecture with 12 transformer layers in the bottleneck was used in [11] for guidewire segmentation. However, the method was evaluated only on 11 image sequences, and instead of segmenting the whole guidewire, the paper only segments the guidewire tip, which is much more visible. Moreover, the method is missing important training details, such as the training loss function, data augmentation information, and there is no code available, which may hinder reproducibility. The authors’ follow-up paper [12] introduces a background residual attention layer and multiple frames to obtain even better guidewire tip segmentation results, but the paper has the same reproducibility issues.

Another vision transformer has been used in [13] together with a shape-sensitive loss function to improve the segmentation accuracy for many standard CNN architectures such as UNet [4], TransUNet [14], SwinU-Net [15], etc. This work is complementary to our work since it introduces a loss function, while our work introduces a novel architecture that is not a UNet. The shape-sensitive loss could also be used in principle together with our architecture to further increase accuracy.

From the guidewire segmentation papers discussed in this section, one could see that the segmentation results vary a lot from dataset to dataset. This is probably due to the variability introduced by the fluoroscopy machines, X-ray intensity, sensor sensitivity, etc., as well as the quality of the annotation. Therefore, in our opinion, guidewire segmentation methods cannot be compared if they are evaluated on different datasets. They can only be compared on the same dataset using the same evaluation measure. Actually, ref. [16] has pointed out that the same conclusion applies to many other medical image segmentation tasks.

In that respect, ref. [17] introduced the CathAction dataset, a dataset of more than 23,000 X-ray images obtained on endovascular interventions on pigs and phantoms. This dataset, together with a more challenging guidewire dataset, will be used in experiments to evaluate the proposed method and compare it with the state of the art.

### 2.2. Guidewire Localization

A hierarchical method for guidewire localization was introduced in [18]. The method first detects short segments on the guidewire using a trained object detector. The segments are used as nodes in a weighted graph, where the edge weights are obtained by another classifier. Finally, a curve is obtained as the shortest path in the graph; thus, a single curve is obtained for each image. In contrast, the proposed approach uses a deep CNN to obtain a good segmentation, from which initial curves are extracted, which are linked using a matching algorithm and a continuity measure.

Besides the Res-UNet segmentation method described in Section 2.1, ref. [2] also introduced a catheter localization method that extracts a centerline using skeletonization and connected components. The extracted curves are merged into a single curve using heuristics. Our proposed localization method also extracts centerlines using a type of skeletonization, but it constructs the curves as maximal chains of degree two nodes instead of connected components, which ensures that each obtained curve is a chain of pixels with no bifurcations. Moreover, our method uses the Hungarian algorithm and a measure of curve continuation for merging the curves, and the final number of curves is obtained automatically.

The *k*-NN-based method from [6] connects the segmented guidewire blocks using a greedy energy minimization algorithm that tries to minimize the sum of distances and the cosine of angles between the connected blocks.

## 3. Method Description

The proposed guidewire localization method is composed of four steps:

1. **Guidewire segmentation**, which labels the image pixels whether they belong to the guidewire or not.
2. **Initial curve extraction**, which takes the segmentation result and returns a number of pixel chains as initial curves.
3. **Perceptual curve grouping**, which merges the initial curves into longer curves based on a continuation measure.
4. **Cleanup**, an optional step that removes all obtained curves that are shorter than $l_{min}$ in length.

**Figure 1:**
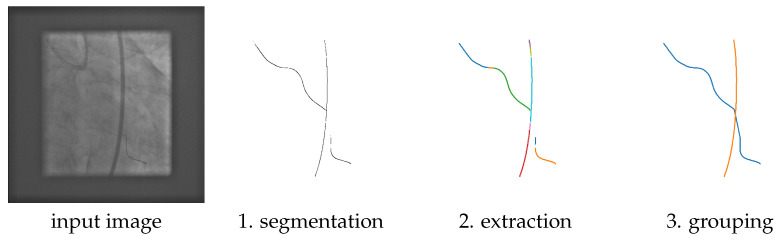
Example output obtained after each step of the proposed method on a test image. Each curve is shown in a different color.

The first three steps are illustrated in Figure 1, and will be described in the following subsections.

### 3.1. Guidewire Segmentation

This step uses a deep CNN to obtain a good guidewire segmentation. The quality of the segmentation will be reflected in the quality of the obtained localization result, which is why we aim for the best possible segmentation.

For that reason, we introduce a novel segmentation method called MSLNet, described below.

#### Proposed MSLNet Segmentation Architecture

**Figure 2:**
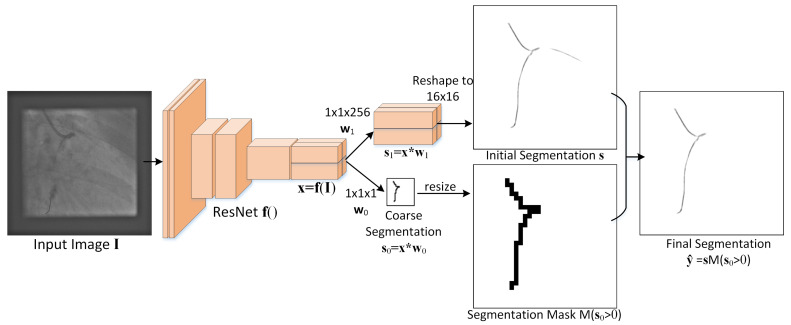
Diagram of the proposed MSLNet guidewire segmentation architecture.

The proposed guidewire segmentation architecture is illustrated in Figure 2. It contains a ResNet (or other type of) feature extractor **f**() and two convolution filters, $w_{0}$ of size 1 × 1 × 1 and $w_{1}$ of size $1\;\times \;1\;\times \;z^{2}$, with z = 16 in our experiments.

The MSLNet segmentation method consists of the following steps:

1. From an image I of size H × W, the ResNet is used as an encoder to extract a feature map x = f(I) of size h × w × C with h = H/z, w = W/z.
2. An initial segmentation s is obtained from the feature map x using the $1\;\times \;1\;\times \;z^{2}$ convolution kernel $w_{1}$, which produces a map $s_{1}$ of size $h\;\times \;w\;\times \;z^{2}$. Each $z^{2}$-dimensional vector from this h × w map is reshaped to a z × z patch and placed at the corresponding location in a h × w grid of patches, which together form the initial segmentation s of size hz × wz = H × W.
3. From the feature map x, a coarse segmentation $s_{0}$ of size h × w is also obtained using the 1 × 1 × 1 convolution kernel $w_{0}$.
4. The final segmentation $\hat{y}$ is obtained as $\hat{y}\;=\;s\cdot M\left(I\left(s_{0}\;>\;0\right)\right)$, where I() is the indicator function and M(U) resizes the input U to make it *z* times larger in each direction, without interpolation, thus
  (1) $$M{\left(U\right)}_{i,j}\;=\;U_{\left\lfloor i/z\rfloor ,\lfloor j/z\right\rfloor }.$$

The whole process is summarized in Algorithm 1 below, where the number of channels used in this paper is C = 3072.

**Algorithm 1** MSLNet Segmentation.   **Input:** Image **I** of size H × W, feature extractor (ResNet) **f**, filters **w**_0_, **w**_1_
   **Output:** Binary segmentation $\hat{y}$ of size H × W
1:Compute x = f(I) of size h × w × C, where h = H/z, w = W/z2:Compute $s_{1}\;=\;x\;*\;w_{1}$ of size $h\;\times \;w\;\times \;z^{2}$ and reshape it to h × w × z × z3:Obtain initial segmentation s of size hz × wz = H × W, by tiling the entries $s_{1}\left(i,j,\cdot ,\cdot \right)$ as z × z patches at positions (iz, jz) in s4:Compute $s_{0}\;=\;x\;*\;w_{0}$ of size h × w5:Obtain final segmentation $\hat{y}\;=\;sM\left(I\left(s_{0}\;>\;0\right)\right)$ with M(U) defined in Equation (1)

Observe that this approach requires the input image dimensions to be divisible by *z*. If that is not the case, the image is padded with zeros to make it divisible.

It is worth noting that this architecture directly predicts the segmentation from the encoded representation x without many decoder layers and without skip connections. This reduces the number of trainable parameters and the depth of the CNN, but faces some overfitting issues that are addressed by the coarse segmentation branch $s_{0}$.

This approach can be thought of as a Marginal Space Learning (MSL) approach [18], where the marginal space is the space of coarse segmentations $s_{0}$, which is $z^{2}\;=\;256$ times smaller than the final segmentation space. Only the z × z patches corresponding to locations where $s_{0}\;>\;0$ are expanded to a fine segmentation; the rest are just set to zero. This is the reason this approach is called MSLNet.

The proposed MSLNet approach is specially designed for segmenting small objects that occupy only a small percentage of the image pixels. For example, the guidewire pixels occupy only about 0.3% of the image pixels. In such cases, standard patch-based and fully convolutional networks might overfit, unless trained with sufficient data. MSLNet is better equipped when the training data are limited by using the coarse layer to predict what parts of the image the segmentation should focus on. The coarse layer, predicting just a binary label for each patch, is less prone to overfitting than the fine layer that predicts the whole segmentation for each patch.

### 3.2. Training the MSLNet

Training is carried out end-to-end using a two-term loss function that encourages a good coarse segmentation $s_{0}$ and a good final segmentation $\hat{y}$. This is in contrast with [10], where the coarse segmentation and the UNet are trained separately.

The trainable parameters consist of the ResNet feature extractor f(I) parameters and the two convolution kernels $w_{0},\;w_{1}$.

Given a training example (I,y) with input I and target binary segmentation y, the coarse target $y^{0}$ is first constructed as a binary indicator for the grid of w × w patches, whether they contain at least one guidewire pixel:

(2) $$y^{0}\left(u,v\right)\;=\;\max_{(i,j),\lfloor i/z\rfloor =u,\lfloor j/z\rfloor =v}y\left(i,j\right)$$

After constructing $y^{0}$, the training loss function for an observation $\left(I,y,y^{0}\right)$ has two parts,

(3) $$L\left(I,y,y^{0}\right)\;=\;L_{c}(f(I)*w_{0},y^{0})\;+\;L_{f}(f(I)*w_{1},y),$$

the coarse segmentation loss $L_{c}\left(s_{0},y^{0}\right)$ and the fine segmentation loss $L_{f}\left(s,y\right)$, where f(I) is the ResNet feature extractor and ‘∗’ is the convolution operator.

Inspired by [3], who combine the Dice and BCE losses, the coarse segmentation loss is the sum

(4) $$L_{c}\left(s,y\right)=L_{DC}\left(s,y\right)+L_{BCE}\left(s,y\right)$$

of the Dice loss and the weighted BCE loss. The Dice loss is

(5) $$L_{DC}\left(s,y\right)=\frac{2\sum_{j}y_{j}\sigma \left(\alpha s_{j}\right)}{\sum_{j}\left(y_{j}\;+\;\sigma \left(\alpha s_{j}\right)\right)},$$

where the sums are taken over the coarse pixels, the function σ(x) = 1/(1 + exp(−x)) is the sigmoid, and α is a tuning parameter (α = 50 in our experiments).

The weighted binary cross-entropy (BCE) loss is

(6) $$L_{BCE}\left(s,y^{0}\right)\;=\;-\frac{1}{|y^{0+}|}\;\sum_{\;j\in y^{0+}}\;\log\sigma \left(s_{j}\right)\;+\;\frac{1}{|y^{0-}|}\;\sum_{\;j\in y^{0-}}\;\left(s_{j}-\log\sigma \left(s_{j}\right)\right),$$

where $y^{0+}\;=\;\left\{j,y_{j}^{0}\;=\;1\right\}$ are the positive pixels of the coarse target $y^{0}$ and $y^{0-}\;=\;\left\{j,y_{j}^{0}\;=\;0\right\}$ are the negative ones.

The fine segmentation loss is also the sum of the Dice loss and the weighted binary cross-entropy (BCE) loss:

(7) $$L_{f}\left(s,y\right)\;=\;L_{DC}\left(s,y\right)\;+\;L_{BCE}\left(s,y\right),$$

where here $y^{+}\;=\;\left\{j,y_{j}\;=\;1\right\}\cap M\left(y^{0}\right)$ and $y^{-}\;=\;\left\{j,y_{j}=0\right\}\cap M\left(y^{0}\right)$ with M(U) as defined in Equation (1).

By restricting the fine segmentation loss only to patches where $y^{0}>0$, we make sure that the training data are more balanced, since in this case the percentage of foreground pixels is about 7%, as opposed to when considering the entire image, when the percentage of foreground pixels is about 0.3%.

However, due to inaccuracies in the annotation, the BCE fine segmentation loss might not be the best choice because it is not very robust to labeling noise. For that reason, we also experimented replacing $L_{BCE}\left(s,y\right)$ with the Lorenz loss [19]:

(8) $$L_{LOR}\left(s,y\right)=\frac{1}{|y^{+}|}\sum_{j\in y^{+}}\log\left(1+{\left[\rho \left(1-s_{j}\right)\right]}^{2}\right)+\frac{1}{|y^{-}|}\sum_{j\in y^{-}}\log\left(1+{\left[\rho \left(1+s_{j}\right)\right]}^{2}\right),$$

where ρ(x)=max(x,0) is the ReLU and $y^{+},y^{-}$ are the same as for Equation (7). This loss is more robust to labeling noise because it penalizes a mistake less than the BCE loss.

### 3.3. Initial Curve Extraction

To extract the initial curves, the thresholded segmentation result is processed using the thinning morphological operation so that each pixel of the obtained output has a small number of neighbors, enabling the extraction of the initial curves as pixel chains. Thinning [20] is an iterative morphological algorithm that is applied to a binary image until convergence and aims to find the centerline of a strip of pixels. In our experiments, we used Matlab’s bwmorph with the thinning option and scikit-image’s thin with identical results. We also experimented with two other related morphological operations: skeletonization and medial axis, but observed that thinning obtained slightly better results.

To extract the pixel chains as curves, first the 8-neighbor graph G=(V,E) is constructed with *V* as the positive pixels of the thinned segmentation. On the thinned segmentation result, most nodes of this graph have degree 2, and some have degree 3. Nodes with degrees more than 3 are very rare.

The rest of the curve extraction is described in Algorithm 2 below.

**Algorithm 2** Initial Curve extraction.  **Input:** Binary segmentation $\hat{y}$
  **Output:** Set of initial curves *S*1:Apply morphological thinning to $\hat{y}$, obtaining output t2:Construct the 8-neighbor graph G=(V,E) with V={(i,j),t(i,j)=1}3:Initialize curve set S=Ø4:**while** exists node i∈V of degree 2 **do**5:    Let C=(j,i,k) where j,k are the two neighbors of *i*6:    **while** *k* has degree 2 **do**7:        **if** exists neighbor $k_{1}$ of *k*, $k_{1}\notin C$ **then**8:            $C=(C,k_{1})$9:            Set $k=k_{1}$10:       **end if**11:    **end while**12:    **while** *j* has degree 2 **do**13:        **if** exists neighbor $j_{1}$ of *j*, $j_{1}\notin C$ **then**14:            $C=(j_{1},C)$15:            Set $j=j_{1}$16:        **end if**17:    **end while**18:    Add *C* to *S*: S=S∪{C}19:    Remove from *V* all nodes in *C*: V=V−C, and remove the corresponding edges from *E*.20:**end while**

Lines 6–17 extract the initial curves as maximal chains *C* containing a node *i* of degree 2. Observe that because it is a chain, each curve *C* induces an ordering of its nodes, an ordering that is unique up to its reversal.

### 3.4. Perceptual Curve Grouping

Perceptual curve grouping takes the curves extracted in Section 3.3 and merges them into longer curves using a continuation measure. When two curves are merged, the pixel ordering for one of them might need to be reversed to obtain a consistent ordering for the merged curve. The whole perceptual grouping algorithm is described in Algorithm 3, with its components being described below.

In Algorithm 3, end curve directions $d_{i}$ are estimated using PCA for each curve, and are used for the curve continuation measure.

**Figure 3:**
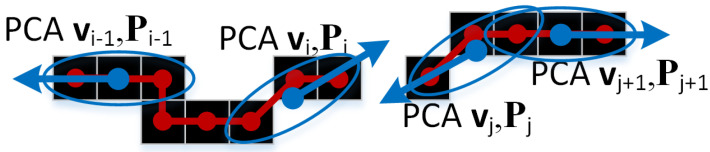
The end curve models are PCAs constructed from the first $n^{pts}$ and last $n^{pts}$ pixels of each curve (shown as blue ellipses) and aligned to point outwards from the curve.

Therefore, for *n* curves, there are 2n PCA models, with models 2i−1 and 2i corresponding to curve i∈{1,…,n}. Model 2i−1 is built from the first *k* points of the curve, as illustrated in Figure 3, while model 2i is built from the last *k* points. If the curve is less than *k* points long, the PCA models are estimated from all curve points. We used k=10 in experiments.

The directions are then aligned in step 6 to point outwards from the curve by making them point towards the respective end of the curve. To align a direction d with mean v to point towards p, first d·(p−v) is computed. If d·(p−v)≥0, then d is already aligned. If d·(p−v)<0, then the direction d is reversed: d←−d.

The point-direction pairs $\left(v_{i},d_{i}\right),\left(v_{j},d_{j}\right)$ are checked in line 10 to be within a distance range $l=\;∥v_{i}-v_{j}∥\;\in \left[1,d_{max}\right]$ and an angle alignment. The angle alignment checks that the angles between $d_{i}$ and $(v_{j}-v_{i})/l$, and between $-d_{j}$ and $(v_{j}-v_{i})/l$, are less than ≈45°, corresponding to $\rho =0.7\approx 1/\sqrt{2}$ in line 10 of Algorithm 3.

**Algorithm 3** Perceptual Curve Grouping (PCG).  **Input:** Curve set $S=\{C_{1},\ldots ,C_{n}\}$, parameters $n^{it},n^{pts},d_{max},\rho ,\tau ,l_{min}$1:**for** t=1 to $n^{it}$ **do**2:    **for** i=1 to *n* **do**3:        Let $\left(p_{1},\ldots ,p_{l}\right)$ be the points of curve $C_{i}$4:        $v_{2i-1},d_{2i-1}=\mathrm{PCA}(\left\{p_{j},j=1:n^{pts}\right\},1)$5:        $v_{2i},d_{2i}=\mathrm{PCA}(\left\{p_{j},j=l-n^{pts}:l\right\},1)$6:        Align $d_{2i-1},d_{2i}$ with $p_{1}$ and $p_{l}$, respectively7:     **end for**8:     **for** $\left(i,j\right)\in {\left\{1,\ldots ,2n\right\}}^{2}$ **do**9:         Set $l=\;∥v_{i}-v_{j}∥$ and $u^{x}=\left(v_{j}-v_{i}\right)/l$10:       **if** $\left(l\geq 1\right)\wedge \left(l\leq d_{max}\right)\wedge \left(d_{i}\cdot u^{x}>\rho \right)\wedge \left(d_{j}\cdot u^{x}<-\rho \right)$ **then**11:           $\left(a,b,c)=\mathrm{FitPoly}(d_{i}\cdot u^{y},-d_{j}\cdot u^{y},l\right)$, with $u^{y}=\left(-u_{y}^{x},u_{x}^{x}\right)$12:            $M_{ij}=\int_{0}^{l}{\left(2b+6cx\right)}^{2}dx$13:        **else**14:            $M_{ij}=10^{5}$15:       **end if**16:   **end for**17:   Use the Hungarian algorithm to find a permutation $\sigma ={\mathrm{argmin}}_{\sigma }\sum_{i}M_{i\sigma_{i}}$18:   Set $\sigma_{i}=0$ for all *i* such that $M_{i\sigma_{i}}>\tau$19:   v=Validate(σ)20:   S=MergeCurves(S,v)21:**end for**

**Figure 4:**
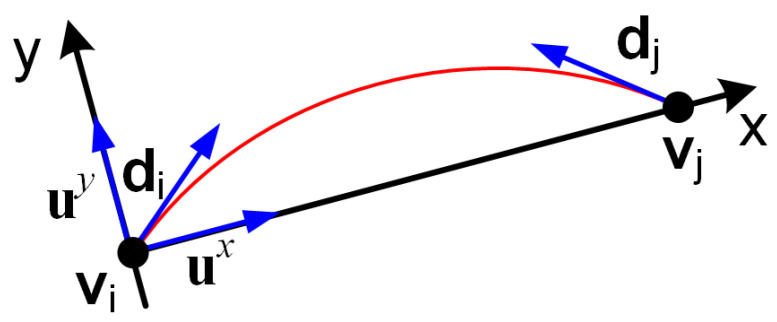
A degree 3 polynomial $f\left(x\right)=ax+bx^{2}+cx^{3}$ is fitted between the points $v_{1}$ and $v_{2}$ on the coordinate system $\left(u^{x},u^{y}\right)$ with the *x*-axis connecting the two points.

For the pairs that pass the check, a continuation measure is computed as $M_{ij}=\int_{0}^{l}{\left(f\prime \prime \left(x\right)\right)}^{2}dx=\int_{0}^{l}{\left(2b+6cx\right)}^{2}dx$ based on fitting a degree 3 polynomial $f\left(x\right)=ax+bx^{2}+cx^{3}$, as specified in Algorithm A1 and illustrated in Figure 4.

For that, a coordinate system is constructed, centered at $v_{i}$ with *x*-axis towards $v_{j}$, thus the *x*-axis is $u^{x}=\left(v_{j}-v_{i}\right)/l$ and the *y*-axis is $u^{y}=\left(-u_{y}^{x},u_{x}^{x}\right)$.

Then a degree three polynomial $f\left(x\right)=ax+bx^{2}+cx^{3}$ is fitted analytically to go through $v_{i},v_{j}$ and be tangent to $d_{i},d_{j}$ as described in Algorithm A1. One can easily check that $M_{ij}=M_{ji}$, so the continuation matrix *M* is symmetric.

The curve ends are matched using the Hungarian algorithm [21] and the matches with cost $M_{i\sigma_{i}}>\tau$ are discarded.

The matches are validated so that only pairs (i,j) such that *i* is matched to *j* and *j* is matched to *i* are kept, as described in Algorithm A2. This step is essential, since the curve merging step would fail without it.

Then the curves are merged based on the validated endpoint matches, as described in Algorithm 4. The function R(C) reverses the points of a curve *C*.

**Algorithm 4** MergeCurves. **Input:** Curves $S=(C_{1},\ldots ,C_{k})$, validated closest index vector $v=(v_{1},\ldots ,v_{2k})$
 **Output:** Merged curves *O*1:Initialize to do set $T=\{i,v_{i}=0\}$ and O=Ø2:**while** |T|>0 **do**3:   Select j∈T and set T=T−{j}4:   Set G=Ø5:   **while** j>0 **do**6:      **if** (j+1)mod2=0 **then**7:          **if** G≠Ø **then**8:             Set $G=(G,C_{i})$ where i=(j+1)/29:          **else**10:           Set $G=C_{i}$ where i=(j+1)/211:         **end if**12:         Set l=j+113:      **else**14:         **if** G≠Ø **then**15:            Set $G=(G,R\left(C_{i}\right))$ where i=j/216:         **else**17:            Set $G=R(C_{i})$ where i=j/218:         **end if**19:         Set l=j−120:      **end if**21:      Set $j=v_{l}$22:   **end while**23:   T=T−{l}24:   Add curve *G* to *O*: O=O∪{G}25:**end while**

## 4. Experiments

Experiments are performed on two datasets: a guidewire dataset and the CathAction dataset [17].

**Figure A1:**
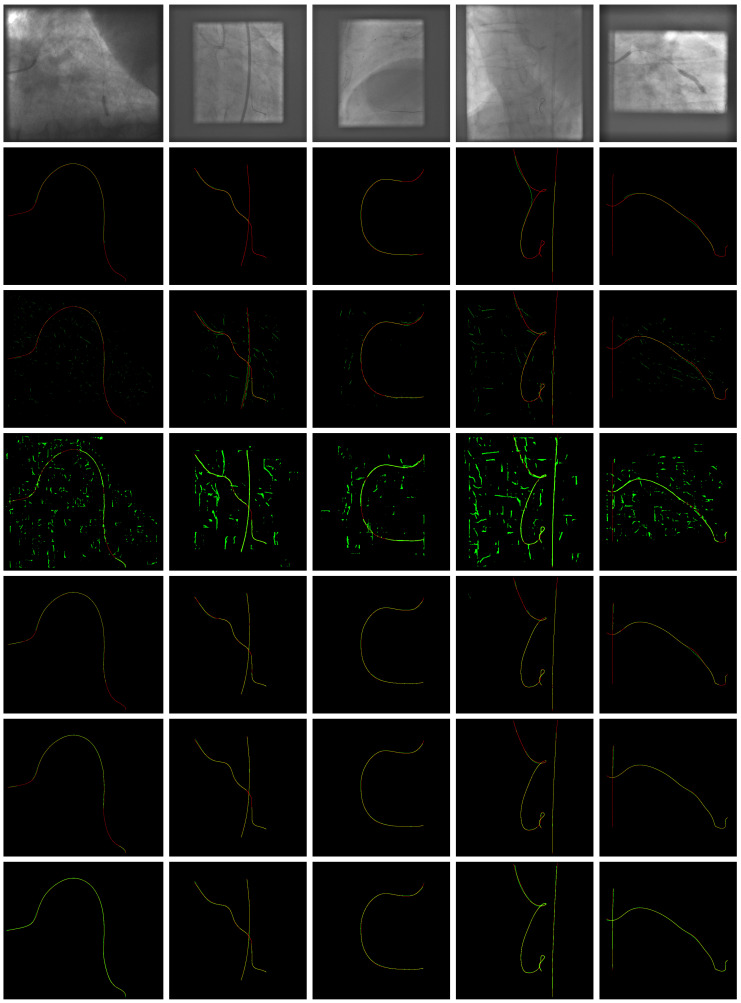
Example segmentation results (green) and annotation (red) on the guidewire test set. From top to bottom: input images, Hierarchical [18], SCNN [8], Two-Phase [10], Res-UNet [2], nnU-Net [3] and MSLNet-Lor.

The guidewire dataset contains 82 fluoroscopic video sequences recorded during coronary angioplasty procedures with 871 frames of various sizes in the range [512,960]×[512,1024]. Of the 82 video sequences, 42 were used as training data, containing 433 frames, and 40 as test data, containing 438 frames. Two example images from this dataset are shown in Figure 1 and Figure 2. The guidewire was annotated using splines, so the accuracy is quite good, usually within 1 pixel of the guidewire. However, in places where the guidewire is not visible or in high curvature places, the annotation might be more than 1 pixel away. Examples of annotation can be seen as red curves in Figure A1, Figure A3 and Figure A5. There is one annotation for each image, and the annotator might be different for different images.

**Figure 5:**
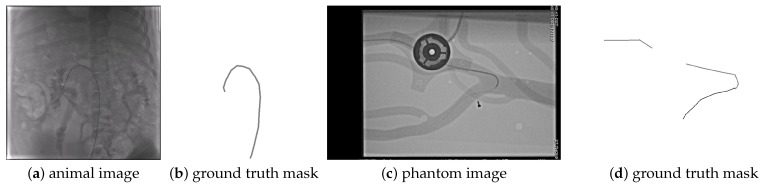
Example images from the CathAction dataset and their corresponding ground truth masks.

**Figure 6:**
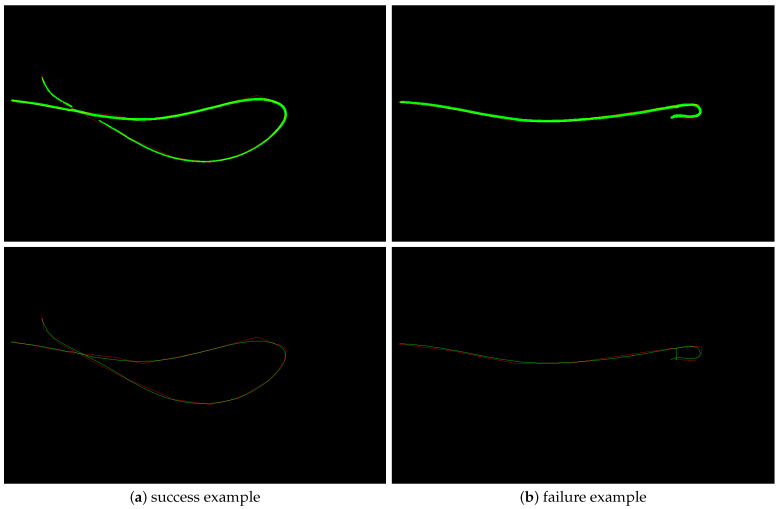
(**a**): a success example of the perceptual grouping method where four curves were connected correctly. (**b**): a failure example. The segmentation result (green) is shown in the top image, and the obtained perceptual grouping result is shown in the bottom image. The annotation is shown in red.

**Figure A2:**
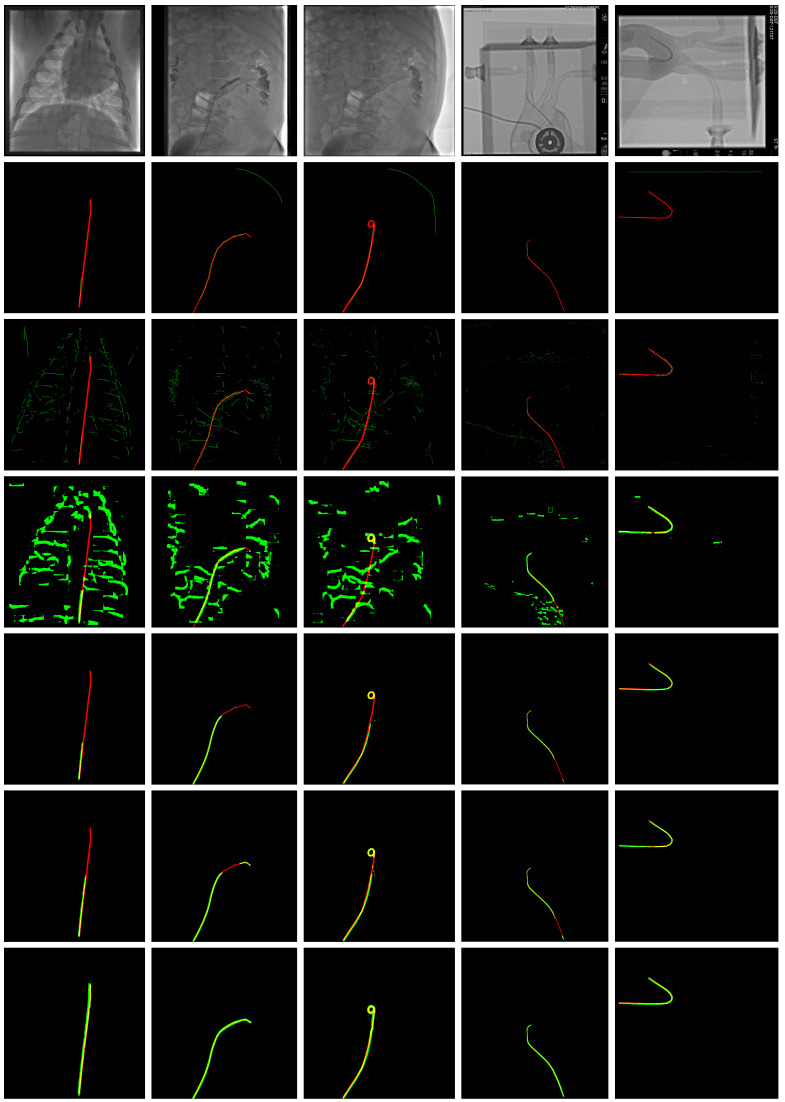
Example segmentation results (green) and annotation (red) on the CathAction test set. From top to bottom: input images, Hierarchical [18], SCNN [8], Two-Phase [10], Res-UNet [2], nnU-Net [3] and MSLNet-Lor.

The CathAction dataset [17] contains 23,449 X-ray images obtained from endovascular interventions on animals (pigs) and imaging phantoms. The CathAction dataset divides the images into 18,758 training images consisting of 4021 animal and 14,737 phantom, and 4691 test images with 1006 animal and 3685 phantom frames. The dataset is annotated for segmentation, using 3–5 pixels thick and very long line segments, so the annotation is not very precise. The catheter pixels and guidewire pixels are annotated with different labels, with examples shown in Figure 5, Figure 6, Figure A2 and Figure A4. As one can see, the guidewire is not annotated inside the catheter. There is one annotation for each image, and the annotator might be different for different images.

### 4.1. Methods Compared and Implementation Details

For segmentation, we compared our proposed MSLNet segmentation approach with the nnU-Net [3], the ResUNet [2], the SCNN [8], the two-step method from [10], and the hierarchical method from [18].

For localization, we compared with the hierarchical localization [18], and with [2], as these two were the only methods that output parameterized curves.

For nnU-Net, we used the GitHub package nnunetv2 https://github.com/MIC-DKFZ/nnUNet (accessed on 13 October 2025), and trained it on our data using the default parameters (batch size 12 for the guidewire dataset and 4 for the CathAction, weight decay $3\cdot 10^{-5}$, Stochastic Gradient Descent with initial learning rate of 0.01, decreasing to 0 linearly), except the number of epochs was 300. The network was initialized with the default initialization for network layers built in PyTorch 2.8.0, and no early stopping was used. Ensembling was not used for a fair comparison.

The MSLNet was also trained as part of the NN-UNet framework, using the same default parameters discussed above, for better segmentation results. This is because the NN-UNet framework offers a rich array of data augmentation transformations that were proven to be useful in many segmentation applications [16]. The ResNet-152 backbone was initialized with the default weights from PyTorch (weights pretrained on ImageNet 1k).

For the Res-UNet [2] architecture, we used the authors’ code from the GitHub package https://github.com/pambros/CNN-2D-X-Ray-Catheter-Detection (accessed on 13 October 2025), but trained it within the NN-UNet framework, again for better segmentation results. The same training parameters were used as for MSLNet and nnU-Net. We also used the authors’ curve grouping code from the same GitHub package to obtain the localization results.

Because the two-step method [10] does not have code available, we used our own implementation of the classification CNN and the segmentation UNet based on the description in the paper. However, we encountered overfitting issues when training these models without data augmentation. For the UNet, without data augmentation, the train $F_{1}$ was 0.78 and the test was 0.26 on the guidewire dataset. With data augmentation, the train $F_{1}$ was 0.36 and the test was 0.26. For the binary classification CNN, data augmentation in the form of random translation up to 4 pixels and random rotation up to 15 degrees helped with overfitting, obtaining a train $F_{1}$ of 0.90 and a test $F_{1}$ of 0.46 on the guidewire dataset.

For the SCNN [8], we used our own implementation of a four-layer SCNN, and for the Hierarchical method [18], we used a pretrained model.

All experiments were performed on a Core I7 computer with 32 GB RAM and a NVIDIA MSI Gaming GeForce 3090 GPU.

The training and test times for the different methods on the guidewire dataset are summarized in Table 1, where the test times and the FLOPS are shown for 512 × 512 images. From Table 1, one could see that the MSLNet has the smallest detection time, due to the fact that the ResNet feature extractor is a fully convolutional network, so that it can be applied directly to images of any size. The other competitive methods, such as NN-UNet and Res-UNet, need to crop images of a certain size on which to apply the segmentation, and then to merge the obtained results into a final segmentation output, which increases the segmentation time.

### 4.2. Evaluation Measures

The methods are evaluated using precision, recall, $F_{1}$ scores, Dice coefficient, IOU (Intersection over Union), and average Hausdorff distance (AHD). Because some methods only obtain a segmentation, separate comparisons are conducted for segmentation and for localization. All results are shown as the average and standard deviation obtained from four independent runs, except for the Hierarchical method from [18], for which we only have a pretrained model.

Annotating a one-pixel-wide guidewire is prone to inaccuracies, which can drastically affect standard measures such as the Dice coefficient $D\left(A,B\right)=\frac{2|A\cap B|}{\left|A|+|B\right|}$ or IOU $I\left(A,B\right)=\frac{\left|A\cap B\right|}{\left|A\cup B\right|}$. To see that, one can imagine evaluating a perfect 1-pixel wide result with a 1-pixel wide annotation that is one pixel off everywhere. Such a result would have a Dice and IOU of 0, while being visually close to perfect. Our conclusion is that Dice and IOU are very good for evaluating blob-like structures, such as organs, but not for very thin structures, such as the guidewire. The catheter segmentation evaluation is somewhere in the middle, because the catheter is 3–5 pixels wide, so the Dice and IOU are less sensitive than for the guidewire evaluation, but they are still sensitive to some extent.

For this reason, besides the Dice and IOU, we will also evaluate using precision, recall, and $F_{1}$ scores, measures that are specifically designed for robustness to such inaccuracies. The precision is defined as the percent of detected pixels that have an annotated guidewire or catheter pixel at a distance of at most 3 pixels. The recall is defined as the percent of guidewire pixels that are at a distance of at most 3 from a detected pixel. The $F_{1}$ score is defined as usual, $F_{1}=2\frac{pr}{p+r}$, in terms of the precision *p* and the recall *r* defined above.

We will also compute the average Hausdorff distance (AHD), which is the average of two measures: the average distance of the detected pixels to the closest annotation pixels, and the average distance of the annotation pixels to the closest detected pixels. This measure is more lenient to annotation inaccuracies, but is not well defined for images where there are no detected pixels.

On the CathAction dataset, the mask annotation is usually several pixels thick, but the method from [18] always outputs a one-pixel-wide result, so the Dice and IOU results are even less relevant for this method on this dataset.

For guidewire localization, the same precision and recall measures are used to evaluate the rasterization of the obtained curves. However, for the CathAction dataset, which does not provide a one-pixel-wide annotation but a several-pixel-wide segmentation, the annotation is first thinned to approximate the location of the guidewire inside the catheter, and this thinned segmentation is used for evaluation. The localization Dice, IOU, and AHD are also evaluated on this thinned segmentation to be able to compare one-pixel-wide results with one-pixel-wide annotations. The guidewire localization is also evaluated on the average number of curves obtained per image, which is desired to be close to the true average number of curves obtained from the annotation, which, on the guidewire test set, is 1.1. We do not know the average number of curves on the CathAction dataset because the dataset does not provide curve annotations, only segmentation masks. Approximating the number of curves using connected components on the CathAction GT masks, we obtained an average of 1.4 curves per image on the test set. However, this might not be an accurate number, as one could see in Figure 5d, where there is only one catheter but the GT mask is broken into two connected components.

### 4.3. Segmentation Results

The segmentation results are displayed in Table 2 for both datasets. Two-sample *t*-tests based on the results of the four independent runs were conducted to compare the best results with the other ones, except the Hierarchical method [18], which is quite behind anyway. Based on these *t*-tests, the best results and the ones that are not significantly worse (p>0.05) are shown in bold.

Two MSLNet versions are shown, with “MSLNet” being trained with the Dice + BCE loss function from Equation (7), and “MSLNet-Lor” being trained with the Dice + the Lorenz loss from Equation (8). 95% confidence intervals for the results from Table 2 are shown in Table A1.

From Table 2, MSLNet performs better than the other methods, including the nnU-Net. The Lorenz loss has a strong influence on the results for the guidewire dataset, where the wire is one pixel wide, increasing the $F_{1}$ score from 89.38 to 92.68, but not for the CathAction dataset, where the catheter is 3–5 pixels wide.

As expected, the nnU-Net [3] performed very well on both datasets, being the second best after MSLNet, followed by the Res-UNet [2]. The other three methods, SCNN [8], Two-phase [10], and Hierarchical [18], are behind by a large margin. The Steerable CNN [8] was designed to only serve as an initial step towards segmentation, using 25×25 patches to predict whether the center pixel is on the guidewire or not. For that reason, it is not capable of capturing long-range interactions, and it has $F_{1}$ scores comparable with the Two-phase method [10], which uses 32×32 patches. Also, because it uses a Spherical Quadrature Filter [22] response map as a preprocessing step, the output is one pixel thin, so the Dice/IOU scores for the CathAction data are very small and unreliable.

We can also see from Table 2 that some methods reach quite high $F_{1}$ scores around 90% while the Dice/IOU scores are very low. In our opinion, this serves as a confirmation that the Dice/IOU scores are more sensitive to annotation inaccuracies for the wire-like structures than the precision/recall and $F_{1}$ scores defined in Section 4.2. Moreover, we see that the Dice/IOU scores are higher on the CathAction data than on the guidewire data for some methods with similar $F_{1}$ scores of about 92%. This is in line with the fact that the catheter that is evaluated in the CathAction data is thicker than the guidewire, so the Dice/IOU are less sensitive to annotation errors for the catheter than for the guidewire. Nevertheless, all four measures ($F_{1}$ score, Dice, IOU, and AHD) tell the same story about how the best segmentation methods compare with each other.

In Table 3 are shown cross-dataset segmentation results, of the models trained on the guidewire dataset and tested on the CathAction data, and vice-versa, with 95% confidence intervals shown in Table A1. The testing on CathAction was separated into the animals and phantoms data, because the images are very different, with the animal images resembling the guidewire data.

From Table 3, one could see that all four methods trained on the guidewire data and tested on the CathAction animals data performed quite well, with Res-UNet, nnU-Net, and MSLNet performing better in terms of $F_{1}$ score and AHD, and MSLNet-Lor better in terms of Dice and IOU. On the CathAction phantoms, none of the methods performed well, with MSLNet-Lor being the best in $F_{1}$ score, Dice, and IOU, and Res-UNet and MSLNet being better in AHD.

Training on the CathAction and testing on the guidewire data yielded quite poor results for all methods, a sign that the CathAction data are easier than the guidewire data. In this case, MSLNet was in the top-performing group on all measures, and nnU-Net was in the top group for $F_{1}$, Dice, and IOU. MSL-Net was in the top group for $F_{1}$ and AHD.

In conclusion, the cross-dataset experiments reveal that the two MSLNet versions performed very well, with MSLNet being in the top group for $F_{1}$ and AHD on all datasets.

### 4.4. Localization Results

The localization results are shown in Table 4, with confidence intervals in Table A2. From Table 4, one could see that the two MSLNet versions obtain the best results in all measures by a large margin, followed by Res-UNet [2] and then by the Hierarchical method [18].

**Figure A3:**
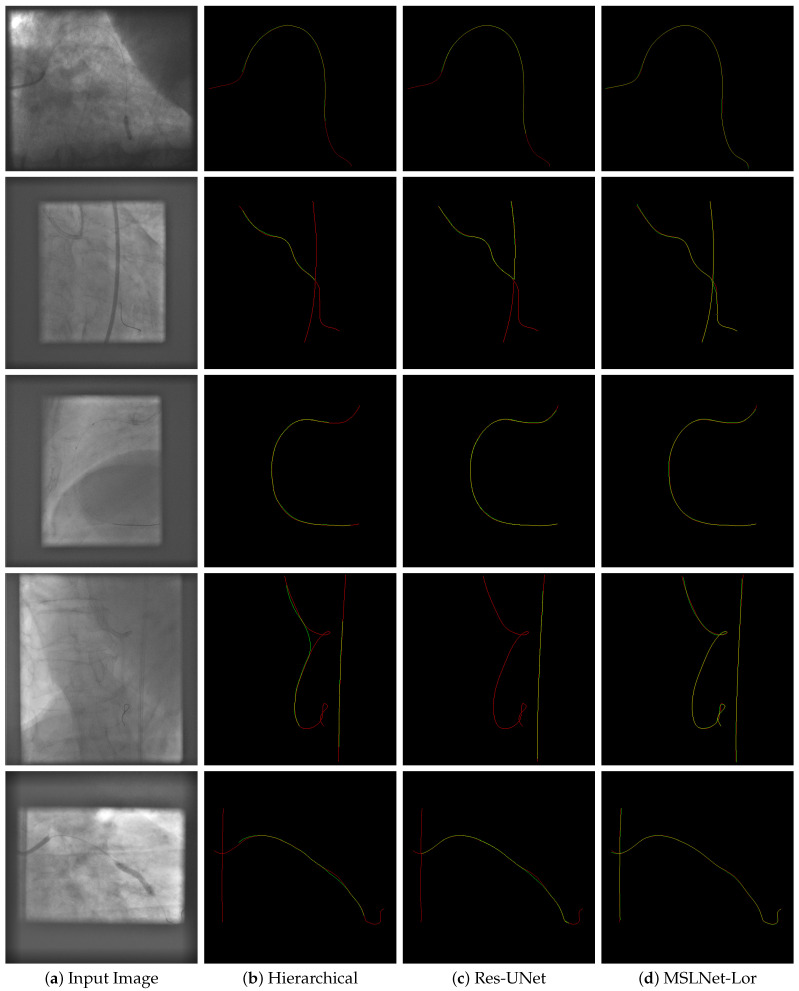
Example localization results (green) and annotation (red) on the guidewire test set.

**Figure A4:**
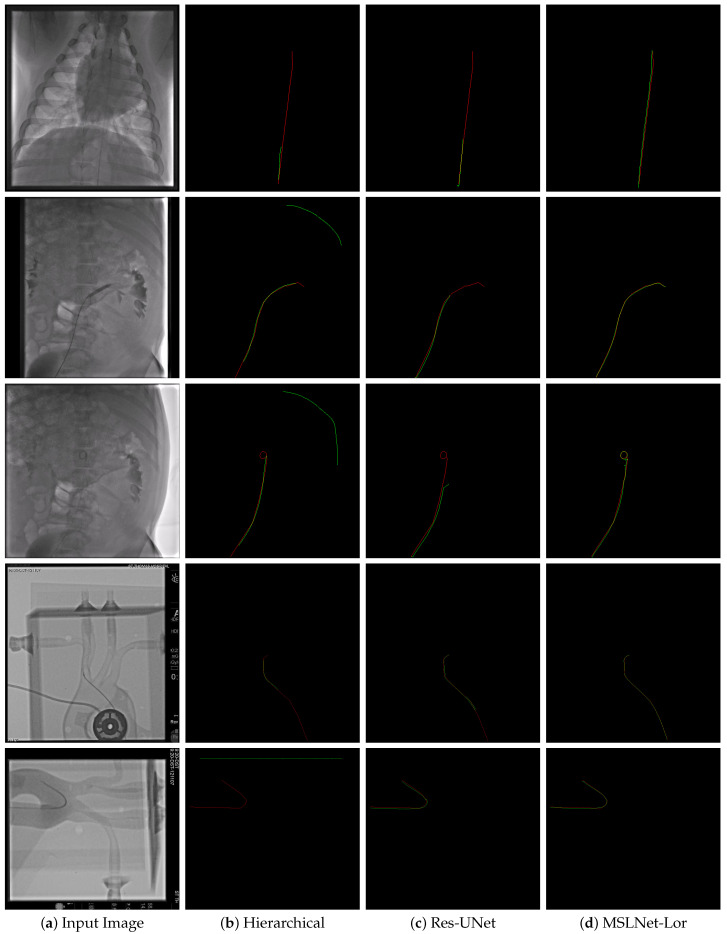
Example localization results (green) and annotation (red) on the CathAction test set. The annotation was thinned, as mentioned in Section 4.2.

Here, the Dice and IOU scores are even less reliable because the results as well as the annotations are both 1-pixel wide (see examples in Figure A3 and Figure A4), and the Dice/IOU scores are extremely sensitive to even 1-pixel discrepancies between the annotation and the localization result.

Looking at the CathAction $F_{1}$ scores, we notice that the Res-UNet [2] curve grouping method starts with a good segmentation $F_{1}$ score of 92.48, and obtains a localization $F_{1}$ score of 53.49, while the MSLNet-Lor starts from a slightly smaller $F_{1}$ score of 92.26, obtaining a localization $F_{1}$ of 83.44. A similar but not so dramatic phenomenon is observed on the guidewire dataset. This is an indication that our proposed perceptual grouping method does a better job at grouping curves based on good continuation than the method from [2].

The perceptual grouping does a very good job in grouping curve fragments, with one such example shown in Figure 6a. We could only find very few failure cases of the perceptual grouping method, but one such case is shown in Figure 6b.

### 4.5. Evaluation of F*_1_* Pixel Tolerance

**Figure 7:**
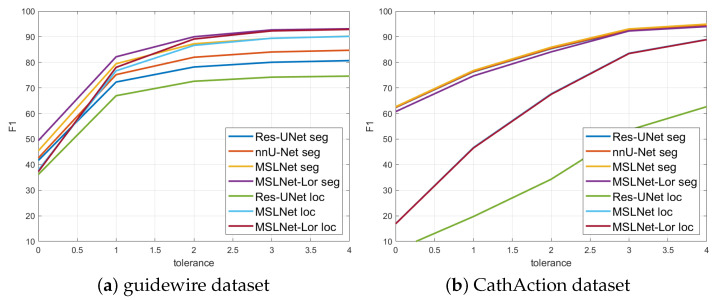
$F_{1}$ values vs. distance tolerance.

The precision, recall, and $F_{1}$ measures have been evaluated using a 3-pixel tolerance. In Table 5 and Figure 7 are shown the test set $F_{1}$ values computed using 0–4 pixel tolerances for the top methods: Res-UNet, nnU-Net, MSLNet, and MSLNet-Lor for segmentation and Res-UNet, MSLNet, and MSLNet-Lor for localization. From Table 5 and Figure 7, one could see that the $F_{1}$ values for 0-pixel tolerance are very small, and they quickly rise with the tolerance distance up to 3 pixels. However, the rise from 3 to 4-pixel tolerance is not so large, which gives us a justification for using the 3-pixel tolerance in our evaluations.

### 4.6. Segmentation Ablation Studies

The segmentation ablation studies evaluate the importance of using the MSL training, the form of the loss function for the coarse segmentation $s_{0}$, and the form of the loss function for the fine segmentation.

**The importance of MSL.** In this experiment, we removed the coarse segmentation $s_{0}$ and its coarse loss function, so we just kept the upper path in Figure 2, so the final segmentation $\hat{y}$ is the initial segmentation s. The results using the Dice + BCE loss from Equation (7), with and without MSL, are shown in Table 6. From Table 6, one could see that the MSL training is important for the guidewire dataset, but not for the CathAction dataset.

**Figure A5:**
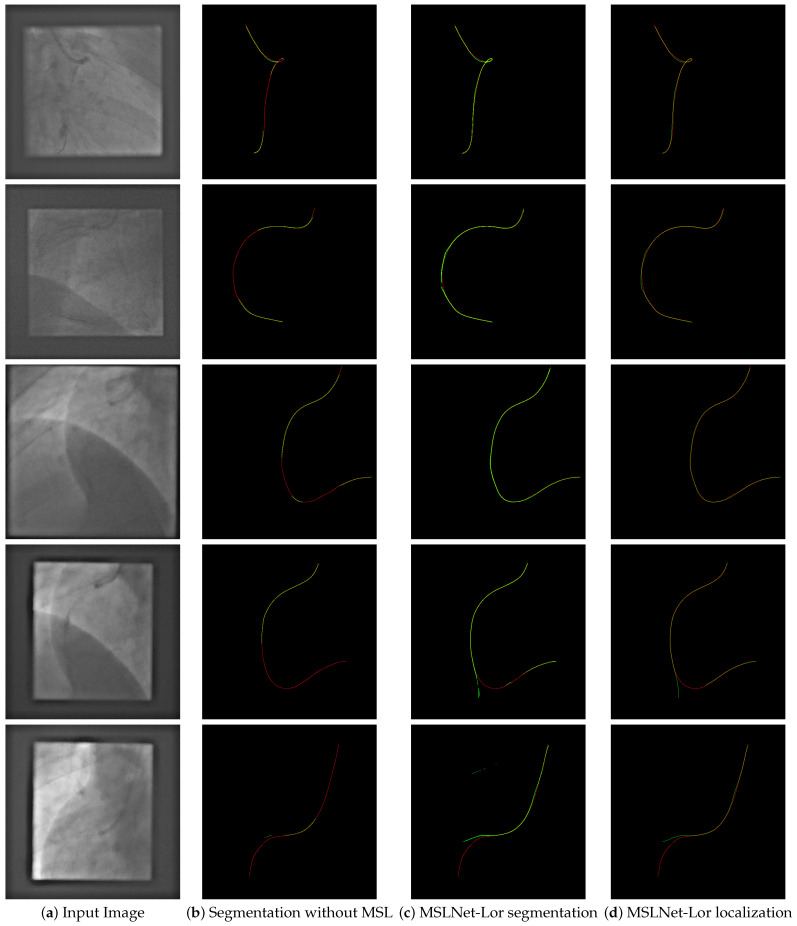
Example results (green) and annotation (red) on the guidewire test set. Shown are input images (**a**), segmentation results without MSL (**b**), with MSL and Lorenz loss (**c**), and localization results (**d**).

Qualitative examples of the architecture without MSL and the MSL-Net Lor segmentation and localization are shown in Figure A5.

**The form of the coarse segmentation loss function $L_{c}\left(s,y\right)$.** Intuitively, the coarse segmentation loss function should follow the pattern observed by [3], that the Dice + BCE loss is better for segmentation than the individual Dice or BCE losses. Indeed, Table 7 confirms this intuition, with the Dice + BCE loss obtaining higher $F_{1}$ scores than using the Dice or BCE losses for the guidewire dataset. However, the Dice scores are higher when using the Dice loss only, because in this case, the Dice is explicitly maximized by that loss. However, the higher Dice score is not reflected in a higher $F_{1}$ score, so we consider it unreliable.

**The form of the fine segmentation loss function $L_{f}\left(s,y\right)$.** For the fine segmentation, we know from [3] that Dice + BCE is better than Dice or BCE alone, so we only compare the Dice + BCE from Equation (7) with Dice + Lorenz, where the Lorenz loss is given in Equation (8). The results are given in Table 8. From Table 8, one could see that the Lorenz loss is important for obtaining higher $F_{1}$ and Dice scores for the guidewire dataset, which has 1-pixel wide annotations, but not for the CathAction dataset, which has thicker annotations.

### 4.7. Localization Ablation Studies

The localization ablation evaluates the importance of the whole perceptual grouping algorithm and its tuning parameters in the quality of the localization result.

**The importance of perceptual grouping.** First, we evaluate the importance of the proposed perceptual grouping algorithm. For that, in Table 9 are shown localization results after initial curve extraction, with and without perceptual grouping on the guidewire dataset starting from the MSLNet-Lor segmentation. Also shown are results after the cleanup step of removing short curves (less than $l_{min}=40$ pixels) directly from the extracted curves, without perceptual grouping.

From Table 9, one could see that the extracted initial curves are broken into many pieces, and just removing the short pieces results in a worse localization result with more curves than when using the proposed perceptual grouping method.

We can also see that cleanup after perceptual grouping has a minimal influence, and the cleanup step can be removed.

**Tuning parameters.** The perceptual grouping method has a number of tuning parameters: the number of iterations $n^{it}$, the number of points to estimate the endpoint directions $n^{pts}$, the maximum distance $d_{max}$ for endpoint matching, the minimum alignment parameter ρ, the maximum continuity score τ and the minimum final curve length $l_{min}$. They are set to the following values: $n^{it}=3,n^{pts}=10,d_{max}=40,\rho =0.7,\tau =10,l_{min}=0$.

The dependence of the obtained result on each of these parameters, while the others are kept to the above values, is shown in Table 10. These experiments are performed on the guidewire dataset starting from the MSLNet-Lor segmentation. From Table 10, one could see that the $F_{1}$ score, AHD, and average number of curves depend only slightly on these parameters when their value is in the range from the table.

**Continuation measure.** The continuation measure between nearby curves used in line 12 of Algorithm 3 is based on fitting a polynomial *f* between the two curves and measuring the $\int_{0}^{l}{\left(f\prime \prime \left(x\right)\right)}^{2}dx$. We also experimented with the Bhattacharyya distance (BD), a measure of similarity between distributions.

For that, in the PCA steps 4–5 of Algorithm 3, when we obtain $v_{i},d_{i},i=1,\ldots ,2n$, we also obtain the corresponding singular values $\lambda_{i}$. Then we use probabilistic PCA (PPCA) models $\Sigma_{i}=d_{i}\lambda_{i}d_{i}^{T}+\sigma^{2}I_{2}$ with $\sigma^{2}=10$ to compute a continuation measure $M_{ij}=BD\left(v_{i},\Sigma_{i},v_{j},\Sigma_{j}\right)$ based on the Bhattacharyya distance (9) instead of lines 11–12 in Algorithm 3. The Bhattacharyya distance for two Gaussians $\left(\mu_{i},\Sigma_{i}\right),\left(\mu_{j},\Sigma_{j}\right)$ is:

(9) $$BD\left(\mu_{i},\Sigma_{i},\mu_{j},\Sigma_{j}\right)=\frac{1}{8}{\left(\mu_{i}-\mu_{j}\right)}^{T}\Sigma^{-1}\left(\mu_{i}-\mu_{j}\right)+\frac{1}{2}\ln\frac{\left|\Sigma \right|}{\sqrt{|\Sigma_{i}\left|\right|\Sigma_{j}|}},$$

where $\Sigma =\frac{\Sigma_{i}+\Sigma_{j}}{2}$. Observe that this BD measure has one more tuning parameter than the polynomial measure: $\sigma^{2}$.

An evaluation of the BD continuation measure with different values of $\rho ,\tau ,\sigma^{2}$ is shown in Table 11. The other parameters were: $n^{it}=3,n^{pts}=10,d_{max}=40,\rho =0.7,\tau =100,\sigma^{2}=10$. From Table 11, one could see that the dependence on the parameters is minimal, but the best $F_{1}$ score is slightly lower than that obtained by the polynomial measure. The difference is significant; the *p* value for a paired *t* test of the difference in $F_{1}$ values between the two results obtained from the same four MSLNet-Lor segmentation runs is p=0.011, which is quite low. This confirms that the polynomial measure $\int_{0}^{l}{\left(f\prime \prime \left(x\right)\right)}^{2}dx$ based on the polynomial fit is better than the BD continuation measure.

## 5. Discussion

The segmentation experiments show that the end-to-end trained segmentation methods obtain better results than other methods that obtain the result in a number of steps that are trained separately. These experiments also indicate that the proposed MSLNet segmentation method obtains competitive results with the other methods evaluated, on both datasets. Moreover, using the Lorenz loss for robustness to annotation imperfections further improves the results for the guidewire dataset, but not for the CathAction data, where the annotation is thicker and the imperfections are not so important.

The localization experiments show that the proposed perceptual grouping method is better than the existing methods evaluated in organizing the segmented pixels into a number of initial curves, together with filling in gaps between the initial curves based on good continuation. The proposed perceptual organization method is less greedy than existing methods because it finds the global minimum of a loss function using the Hungarian algorithm instead of heuristics or greedy loss minimization.

## 6. Conclusions

This paper introduced a method for guidewire localization based on a perceptual grouping algorithm that groups a set of initial curves into longer curves based on a good continuation measure using PCA models at the curve endpoints. The initial curves are extracted from a guidewire segmentation result.

The paper also introduces a guidewire segmentation method based on a ResNet that directly predicts a coarse segmentation as well as a fine segmentation at promising locations indicated by the coarse segmentation.

Experiments on two datasets show that the proposed method obtains competitive results, usually outperforming existing guidewire segmentation and localization methods.

The perceptual organization method has some weaknesses, since it relies on a good guidewire segmentation, and it has six tuning parameters, which could be considered to be too many. However, we saw in the ablation study that the method is quite robust to the tuning parameters, taking a large range of values.

In the future, we plan to study deep-learning-based methods for perceptual grouping that can be trained end-to-end, possibly by reinforcement learning.

## Figures and Tables

**Table 1:** Computation times on the guidewire dataset.

Method	# Pars$\cdot 10^{6}$	Train Time (h)	Test Time (ms)	FLOPS
Hierarchical [18]	1.2	-	724	-
SCNN [8]	4.0	15.5	45	3.3
Two-phase [10]	15.0	22.3	1856	265.2
Res-UNet [2]	14.2	19.3	134	9.7
nnU-Net [3]	126.6	14.3	276	59.9
MSLNet	58.9	18.2	28	60.8

**Table 2:** Segmentation results. Shown are the mean (and std) obtained on the test set from four independent runs. Best results are shown in bold (see text for details).

Method	Precision	Recall	$F_{1}$	Dice	IOU	AHD
Guidewire dataset						
Hierarchical [18]	94.80	57.14	71.30	19.25	11.00	29.1
SCNN [8]	44.86 (1.31)	62.15 (0.78)	52.09 (0.62)	18.67 (0.11)	10.71 (0.06)	36.6 (2.7)
Two-phase [10]	28.18 (2.12)	70.74 (3.59)	40.22 (2.18)	15.45 (1.22)	8.63 (0.77)	36.9 (1.4)
Res-UNet [2]	94.30 (0.99)	69.50 (0.13)	80.02 (0.33)	40.17 (0.26)	26.53 (0.20)	21.1 (0.7)
nnU-Net [3]	**98.18 (0.35)**	73.47 (0.89)	84.04 (0.54)	41.51 (0.67)	27.22 (0.54)	14.0 (0.8)
MSLNet	**98.25 (0.22)**	81.98 (0.45)	89.38 (0.29)	45.06 (0.11)	30.05 (0.10)	10.5 (0.7)
MSLNet-Lor	97.16 (0.24)	**88.60 (0.46)**	**92.68 (0.20)**	**48.82** (0.03)	**33.00 (0.02)**	**7.0 (0.3)**
CathAction dataset						
Hierarchical [18]	56.13	42.23	48.20	11.92	6.55	90.9
SCNN [8]	70.68 (0.19)	70.32 (1.18)	70.50 (0.67)	17.01 (0.26)	9.37 (0.15)	40.9 (1.9)
Two-phase [10]	50.24 (3.57)	93.49 (0.21)	65.28 (2.94)	36.71 (2.15)	23.54 (1.65)	34.6 (5.7)
Res-UNet [2]	**91.33 (0.13)**	93.66 (0.03)	92.48 (0.08)	62.07 (0.05)	46.13 (0.07)	1.7 (0.0)
nnU-Nnet [3]	**91.46 (0.30)**	93.82 (0.47)	92.62 (0.15)	62.19 (0.18)	46.25 (0.20)	1.7 (0.1)
MSLNet	**91.52 (0.10)**	94.62 (0.13)	**93.05 (0.02)**	**62.51 (0.04)**	**46.58 (0.04)**	**1.5 (0.0)**
MSLNet-Lor	87.36 (0.20)	**97.74 (0.06)**	92.26 (0.09)	60.45 (0.14)	44.04 (0.15)	**1.5 (0.0)**

**Table 3:** Cross-dataset segmentation results. Shown are the mean (and std) obtained on the test set from four independent runs. Best results are shown in bold (see text for details).

Method	Precision	Recall	$F_{1}$	Dice	IOU	AHD
Train on CathAction, test on Guidewire						
Res-UNet [2]	69.52 (1.65)	21.29 (0.80)	32.59 (1.11)	16.39 (0.44)	9.40 (0.25)	54.2 (2.1)
nnU-Nnet [3]	**80.01 (5.49)**	**26.36 (4.79)**	**39.51 (5.90)**	**18.57 (2.20)**	**10.56 (1.30)**	53.6 (6.4)
MSLNet	**73.82 (2.21)**	**31.79 (3.53)**	**44.37 (3.77)**	**20.27 (1.28)**	**11.89 (0.80)**	**43.3 (4.0)**
MSLNet-Lor	66.56 (3.16)	**35.82 (6.25)**	**46.42 (5.92)**	17.01 (1.46)	9.71 (0.84)	**36.9 (4.6)**
Train on Guidewire, test on CathAction animals						
Res-UNet [2]	**89.97 (2.64)**	84.15 (0.38)	**86.94 (1.30)**	31.77 (0.36)	19.05 (0.24)	**9.5 (2.8)**
nnU-Nnet [3]	**90.01 (1.60)**	86.41 (0.54)	**88.16 (0.58)**	33.15 (0.95)	20.08 (0.70)	**9.0 (1.1)**
MSLNet	**90.82 (1.86)**	85.25 (0.39)	**87.94 (0.84)**	30.01 (0.39)	17.86 (0.28)	**8.9 (1.6)**
MSLNet-Lor	70.36 (2.01)	**90.73 (0.22)**	79.24 (1.29)	**38.19 (0.67)**	**24.00 (0.51)**	24.9 (2.0)
Train on Guidewire, test on CathAction phantoms						
Res-UNet [2]	**70.59 (1.11)**	50.95 (2.12)	59.15 (1.51)	15.68 (0.77)	8.68 (0.45)	**41.0 (2.8)**
nnU-Nnet [3]	54.15 (4.14)	50.74 (4.39)	52.35 (4.04)	15.24 (1.53)	8.40 (0.90)	85.3 (12.9)
MSLNet	**70.31 (1.75)**	57.52 (3.26)	**63.19 (1.73)**	18.85 (1.42)	10.61 (0.88)	**40.3 (4.1)**
MSLNet-Lor	54.74 (2.82)	**67.97 (0.18)**	**60.60 (1.75)**	**25.75 (0.99)**	**15.12 (0.66)**	72.5 (8.1)

**Table 4:** Localization results. Shown are the mean (and std) obtained on the test set from four independent runs. Best results are shown in bold (see text for details).

Method	Precision	Recall	$F_{1}$	Dice	IOU	AHD	Avg #Curves
Guidewire dataset							
Hierarchical [18]	94.80	57.48	71.56	18.79	10.68	29.1	1.0 (0.0)
Res-UNet [2]	95.66 (0.82)	60.62 (0.75)	74.20 (0.39)	34.30 (0.32)	21.84 (0.27)	35.0 (0.9)	1.0 (0.0)
MSLNet	**97.56** (0.14)	82.52 (0.73)	89.41 (0.41)	**37.29 (0.14)**	**23.74 (0.09)**	10.9 (0.8)	1.3 (0.0)
MSLNet-Lor	96.76 (0.20)	**88.19 (0.49)**	**92.28 (0.22)**	37.00 (0.09)	23.51 (0.06)	7.7 (0.4)	1.3 (0.0)
CathAction dataset							
Hierarchical [18]	49.62	45.67	47.57	8.48	4.61	91.3	1.0 (0.0)
Res-UNet [2]	56.88 (0.45)	50.49 (0.75)	53.49 (0.62)	6.28 (0.12)	3.33 (0.07)	6.3 (0.6)	1.0 (0.0)
MSLNet	**85.65 (0.09)**	81.58 (0.14)	**83.57 (0.04)**	**16.90 (0.07)**	**9.53 (0.04)**	2.6 (0.0)	1.1 (0.0)
MSLNet-Lor	84.93 (0.08)	**82.01 (0.05)**	83.44 (0.06)	**16.88 (0.03)**	**9.51 (0.02)**	2.6 (0.0)	1.3 (0.1)

**Table 5:** $F_{1}$ scores obtained using different distance tolerance thresholds, from 0 to 4 pixels.

Segmentation					Localization		
Distance	Res-UNet [2]	nnU-Nnet [3]	MSLNet	MSLNet-Lor	Res-UNet [2]	MSLNet	MSLNet-Lor
Guidewire dataset							
0	41.66 (0.22)	42.61 (0.66)	45.41 (0.13)	49.42 (0.03)	36.16 (0.26)	37.89 (0.09)	37.29 (0.10)
1	72.30 (0.29)	75.19 (0.57)	79.45 (0.19)	82.16 (0.15)	66.95 (0.40)	76.69 (0.25)	78.04 (0.24)
2	78.17 (0.32)	81.98 (0.54)	87.25 (0.23)	89.99 (0.19)	72.60 (0.40)	86.62 (0.27)	89.10 (0.29)
3	80.02 (0.33)	84.04 (0.54)	89.38 (0.29)	92.68 (0.20)	74.20 (0.39)	89.41 (0.41)	92.28 (0.22)
4	80.67 (0.34)	84.71 (0.55)	90.20 (0.25)	93.11 (0.20)	74.61 (0.40)	90.14 (0.29)	92.86 (0.24)
CathAction dataset							
0	62.32 (0.05)	62.40 (0.16)	62.66 (0.03)	60.73 (0.14)	6.55 (0.12)	16.92 (0.07)	16.90 (0.03)
1	76.30 (0.08)	76.42 (0.18)	76.79 (0.04)	74.69 (0.14)	19.72 (0.34)	46.70 (0.07)	46.51 (0.10)
2	85.35 (0.09)	85.49 (0.16)	85.91 (0.03)	84.09 (0.13)	34.31 (0.52)	67.71 (0.07)	67.50 (0.07)
3	92.48 (0.08)	92.62 (0.15)	93.05 (0.02)	92.26 (0.09)	53.49 (0.62)	83.57 (0.04)	83.44 (0.06)
4	94.36 (0.08)	94.52 (0.13)	94.92 (0.01)	93.98 (0.08)	62.68 (0.57)	88.95 (0.02)	88.86 (0.05)

**Table 6:** The influence of using the MSL training for segmentation accuracy.

Dataset	MSL	Precision	Recall	$F_{1}$	Dice	IOU	AHD
Guidewire	N	98.78 (0.14)	78.74 (0.38)	87.63 (0.27)	44.47 (0.39)	29.71 (0.30)	11.5 (0.6)
Guidewire	Y	98.25 (0.22)	81.98 (0.45)	89.38 (0.29)	45.06 (0.11)	30.05 (0.10)	10.5 (0.7)
CathAction	N	91.27 (0.05)	94.80 (0.11)	93.01 (0.07)	62.68 (0.04)	46.76 (0.04)	1.5 (0.0)
CathAction	Y	91.52 (0.10)	94.62 (0.13)	93.05 (0.02)	62.51 (0.04)	46.58 (0.04)	1.5 (0.0)

**Table 7:** The influence of the form of the coarse segmentation loss function $L_{c}\left(s,y\right)$ on the guidewire dataset.

$L_{c}$ Loss	Precision	Recall	$F_{1}$	Dice	IOU	AHD
Dice	97.04 (0.66)	79.84 (0.80)	87.60 (0.70)	45.13 (0.57)	30.14 (0.43)	11.3 (0.9)
BCE	98.14 (0.17)	80.30 (0.57)	88.33 (0.39)	44.19 (0.28)	29.40 (0.21)	10.8 (0.6)
Dice + BCE	98.25 (0.22)	81.98 (0.45)	89.38 (0.29)	45.06 (0.11)	30.05 (0.10)	10.5 (0.7)

**Table 8:** The influence of the form of the fine segmentation loss function $L_{f}\left(s,y\right)$.

Dataset	$L_{f}$ **Loss**	Precision	Recall	$F_{1}$	Dice	IOU	AHD
Guidewire	Dice + BCE	98.25 (0.22)	81.98 (0.45)	89.38 (0.29)	45.06 (0.11)	30.05 (0.10)	10.5 (0.7)
Guidewire	Dice + LOR	97.16 (0.24)	88.60 (0.46)	92.68 (0.20)	48.82 (0.03)	33.00 (0.02)	7.0 (0.3)
CathAction	Dice + BCE	91.52 (0.10)	94.62 (0.13)	93.05 (0.02)	62.51 (0.04)	46.58 (0.04)	1.5 (0.0)
CathAction	Dice + LOR	87.36 (0.20)	97.74 (0.06)	92.26 (0.09)	60.45 (0.14)	44.04 (0.15)	1.5 (0.0)

**Table 9:** Ablation of the two main localization steps after curve extraction: perceptual grouping (PG) and cleanup (removing short curves) on the guidewire dataset. Shown are test set means (std) from four independent runs.

Perceptual Grouping	Cleanup	Precision	Recall	$F_{1}$	Dice	IOU	AHD	Avg #Curves
N	N	97.24 (0.20)	87.76 (0.44)	92.26 (0.20)	37.01 (0.09)	23.52 (0.07)	7.4 (0.3)	2.5 (0.0)
N	Y	97.48 (0.21)	86.61 (0.46)	91.73 (0.25)	36.83 (0.11)	23.39 (0.07)	8.2 (0.5)	1.6 (0.0)
Y	N	96.76 (0.20)	88.19 (0.49)	92.28 (0.22)	37.00 (0.09)	23.51 (0.06)	7.4 (0.3)	1.3 (0.0)
Y	Y	96.88 (0.20)	88.08 (0.53)	92.27 (0.25)	36.98 (0.11)	23.49 (0.08)	7.7 (0.4)	1.3 (0.0)

**Table 10:** The influence of perceptual grouping parameters $n^{it},n^{pts},d_{max},\rho ,\tau$ and $l_{min}$ in localization $F_{1}$ scores and average number of curves per image. The base segmentation is from MSLNet-Lor. Shown are test means (std) from four runs.

$n^{it}$	1	2	3	4
$F_{1}$	92.28 (0.20)	92.31 (0.22)	92.28 (0.22)	92.26 (0.22)
AHD/# curves	7.4 (0.3)/1.5 (0.0)	7.4 (0.3)/1.4 (0.0)	7.4 (0.3)/1.3 (0.0)	7.4 (0.3)/1.3 (0.0)
$n^{pts}$	3	5	10	20
$F_{1}$	92.22 (0.27)	92.10 (0.25)	92.28 (0.22)	92.27 (0.25)
AHD/# curves	7.4 (0.3)/1.4 (0.0)	7.6 (0.3)/1.3 (0.0)	7.4 (0.3)/1.3 (0.0)	7.4 (0.3)/1.4 (0.0)
$d_{max}$	10	20	40	50
$F_{1}$	92.03 (0.31)	92.29 (0.20)	92.28 (0.22)	92.26 (0.22)
AHD/# curves	7.7 (0.4)/1.5 (0.0)	7.4 (0.2)/1.4 (0.0)	7.4 (0.3)/1.3 (0.0)	7.4 (0.3)/1.3 (0.0)
ρ	0.3	0.5	0.7	0.9
$F_{1}$	92.24 (0.23)	92.25 (0.23)	92.28 (0.22)	92.29 (0.22)
AHD/# curves	7.4 (0.3)/1.3 (0.0)	7.4 (0.3)/1.3 (0.0)	7.4 (0.3)/1.3 (0.0)	7.4 (0.3)/1.4 (0.0)
τ	1	3	10	30
$F_{1}$	92.28 (0.22)	92.28 (0.22)	92.28 (0.22)	92.28 (0.22)
AHD/# curves	7.4 (0.3)/1.3 (0.0)	7.4 (0.3)/1.3 (0.0)	7.4 (0.3)/1.3 (0.0)	7.4 (0.3)/1.3 (0.0)
$l_{min}$	0	20	40	60
$F_{1}$	92.28 (0.22)	92.28 (0.22)	92.27 (0.25)	92.23 (0.23)
AHD/# curves	7.4 (0.3)/1.3 (0.0)	7.5 (0.3)/1.3 (0.0)	7.7 (0.4)/1.3 (0.0)	7.8 (0.6)/1.3 (0.0)

**Table 11:** Parameter experiments using the Bhattacharyya distance continuation measure (9). The base segmentation is from MSLNet-L. Shown are test means (std) from four runs.

ρ	0.3	0.5	0.7	0.9
$F_{1}$	92.13 (0.22)	91.92 (0.26)	91.97 (0.28)	92.21 (0.28)
AHD/# curves	7.5 (0.3)/1.5 (0.0)	7.8 (0.3)/1.3 (0.1)	7.7 (0.4)/1.3 (0.0)	7.5 (0.3)/1.4 (0.0)
τ	1	10	100	300
$F_{1}$	91.97 (0.26)	92.01 (0.28)	91.97 (0.28)	91.97 (0.28)
AHD/# curves	7.7 (0.3)/1.5 (0.0)	7.7 (0.4)/1.4 (0.0)	7.7 (0.4)/1.3 (0.0)	7.7 (0.4)/1.3 (0.0)
$\sigma^{2}$	0.1	1	10	100
$F_{1}$	91.27 (0.22)	91.26 (0.23)	91.97 (0.28)	91.43 (0.31)
AHD/# curves	8.5 (0.4)/1.4 (0.0)	8.5 (0.4)/1.3 (0.0)	7.7 (0.4)/1.3 (0.0)	8.3 (0.4)/1.3 (0.0)

## Data Availability

The CathAction dataset is available at https://airvlab.github.io/cathaction/ (accessed on 13 October 2025). The guidewire dataset is not publicly available, and we do not have permission to publish it. The code for the proposed MSLNet and perceptual organization methods is available at https://github.com/barbua/MSLNet (accessed on 13 October 2025).

## Abbreviations

The following abbreviations are used in this manuscript:

BCE	Binary cross-entropy
BD	Bhattacharyya distance
CNN	Convolutional neural network
*k*-NN	*k*-nearest neighbors
MSL	Marginal Space Learning
PCA	Principal Component Analysis
ResNet	Residual network
SCNN	Steerable CNN

## Appendix A

Algorithm A1 fits a polynomial of degree three $f\left(x\right)=ax+bx^{2}+cx^{3}$, as illustrated in Figure 4.

**Algorithm A1** FitPoly.
**Input:** $\alpha_{0},\alpha_{1},l$ **Output:** Degree 3 polynomial $f\left(x\right)=ax+bx^{2}+cx^{3}$ s.t. $f\left(0\right)=f\left(l\right)=0,f^{\prime }\left(0\right)=\alpha_{0},f^{\prime }\left(l\right)=\alpha_{1}$ 1: $a=\alpha_{0},b=-\left(2\alpha_{0}+\alpha_{1}\right)/l,c=\left(\alpha_{0}+\alpha_{1}\right)/l^{2}$

Algorithm A2 is a simple algorithm that only keeps the endpoint matches $c_{i},c_{j}$ such that $c_{i}=j$ and $c_{j}=i$.

**Algorithm A2** Validate.
**Input:** Match index vector $c=(c_{1},\ldots ,c_{2k})$ **Output:** Validated match index vector $v=(v_{1},\ldots ,v_{2k})$ 1: Initialize $v=\left(v_{1},\ldots ,v_{2k}\right),v_{i}=0,i=1,\ldots ,2k$ 2: **for** i=1 to 2k **do** 3: Let $j=c_{i}$ 4: **if** i<j and $c_{j}=i$ **then** 5: Set $v_{i}=j$ and $v_{j}=i$ 6: **end if** 7: **end for**

## Appendix B

In Table A1 are shown 95% confidence intervals (CI) for the segmentation results from Table 2 and Table 3.

**Table A1:** Confidence intervals for the segmentation results from Table 2 and Table 3.

Method	Precision	Recall	$F_{1}$	Dice	IOU	AHD
Guidewire dataset						
SCNN	43.58 46.14	61.39 62.91	51.48 52.70	18.56 18.78	10.65 10.77	33.95 39.25
Two-phase	26.10 30.26	67.22 74.26	38.08 42.36	14.25 16.65	7.88 9.38	35.53 38.27
Res-UNet	93.33 95.27	69.37 69.63	79.70 80.34	39.92 40.42	26.33 26.73	20.41 21.79
nnU-Net	97.84 98.52	72.60 74.34	83.51 84.57	40.85 42.17	26.69 27.75	13.22 14.78
MSLNet	98.03 98.47	81.54 82.42	89.10 89.66	44.95 45.17	29.95 30.15	9.81 11.19
MSLNet-Lor	96.92 97.40	88.15 89.05	92.48 92.88	48.79 48.85	32.98 33.02	6.71 7.29
CathAction dataset						
SCNN	70.49 70.87	69.16 71.48	69.84 71.16	16.76 17.26	9.22 9.52	39.04 42.76
Two-phase	46.74 53.74	93.28 93.70	62.40 68.16	34.60 38.82	21.92 25.16	29.01 40.19
Res-UNet	91.20 91.46	93.63 93.69	92.40 92.56	62.02 62.12	46.06 46.20	1.70 1.70
nnU-Nnet	91.17 91.75	93.36 94.28	92.47 92.77	62.01 62.37	46.05 46.45	1.60 1.80
MSLNet	91.42 91.62	94.49 94.75	93.03 93.07	62.47 62.55	46.54 46.62	1.50 1.50
MSLNet-Lor	87.16 87.56	97.68 97.80	92.17 92.35	60.31 60.59	43.89 44.19	1.50 1.50
Train on CathAction, test on Guidewire						
Res-UNet	67.90 71.14	20.51 22.07	31.50 33.68	15.96 16.82	9.16 9.64	52.14 56.26
nnU-Nnet	74.63 85.39	21.67 31.05	33.73 45.29	16.41 20.73	9.29 11.83	47.33 59.87
MSLNet	71.65 75.99	28.33 35.25	40.68 48.06	19.02 21.52	11.11 12.67	39.38 47.22
MSLNet-Lor	63.46 69.66	29.70 41.95	40.62 52.22	15.58 18.44	8.89 10.53	32.39 41.41
Train on Guidewire, test on CathAction animals						
Res-UNet	87.38 92.56	83.78 84.52	85.67 88.21	31.42 32.12	18.81 19.29	6.76 12.24
nnU-Nnet	88.44 91.58	85.88 86.94	87.59 88.73	32.22 34.08	19.39 20.77	7.92 10.08
MSLNet	89.00 92.64	84.87 85.63	87.12 88.76	29.63 30.39	17.59 18.13	7.33 10.47
MSLNet-Lor	68.39 72.33	90.51 90.95	77.98 80.50	37.53 38.85	23.50 24.50	22.94 26.86
Train on Guidewire, test on CathAction phantoms						
Res-UNet	69.50 71.68	48.87 53.03	57.67 60.63	14.93 16.43	8.24 9.12	38.26 43.74
nnU-Nnet	50.09 58.21	46.44 55.04	48.39 56.31	13.74 16.74	7.52 9.28	72.66 97.94
MSLNet	68.59 72.03	54.33 60.71	61.49 64.89	17.46 20.24	9.75 11.47	36.28 44.32
MSLNet-Lor	51.98 57.50	67.79 68.15	58.89 62.31	24.78 26.72	14.47 15.77	64.56 80.44

The CI is obtained using the equation $CI=\mu \pm 1.96\cdot \sigma /\sqrt{n}$ where (μ,σ) are the mean and standard deviation of the performance measures obtained from *n* runs, with n=4 in this case.

In Table A2 are shown 95% confidence intervals (CI) for the localization results from Table 4.

**Table A2:** Confidence intervals for the localization results from Table 4.

Method	Precision	Recall	$F_{1}$	Dice	IOU	AHD	Avg # Curves
Guidewire dataset							
Res-UNet	94.86 96.46	59.88 61.35	73.82 74.58	33.99 34.61	21.58 22.10	34.12 35.88	1.00 1.00
MSLNet	97.42 97.70	81.80 83.24	89.01 89.81	37.15 37.43	23.65 23.83	10.12 11.68	1.30 1.30
MSLNet-Lor	96.56 96.96	87.71 88.67	92.06 92.50	36.91 37.09	23.45 23.57	7.31 8.09	1.30 1.30
CathAction dataset							
Res-UNet	56.44 57.32	49.76 51.23	52.88 54.10	6.16 6.40	3.26 3.40	5.71 6.89	1.00 1.00
MSLNet	85.56 85.74	81.44 81.72	83.53 83.61	16.83 16.97	9.49 9.57	2.60 2.60	1.10 1.10
MSLNet-Lor	84.85 85.01	81.96 82.06	83.38 83.50	16.85 16.91	9.49 9.53	2.60 2.60	1.20 1.40

In Table A3 are shown segmentation results evaluated at the sequence level on the guidewire test set. That means that the performance measures are first averaged over each sequence and then these obtained averages are averaged over the 40 test sequences. The corresponding localization results evaluated at the sequence level are shown in Table A4.

**Table A3:** Segmentation results evaluated at the sequence level. Shown are the mean (and std) obtained on the guidewire test set from four independent runs.

Method	Precision	Recall	$F_{1}$	Dice	IOU	AHD
Trained on the Guidewire dataset						
Hierarchical	92.27	60.24	72.89	19.72	11.29	23.9
SCNN	17.67 (8.70)	82.05 (7.44)	27.62 (12.37)	10.81 (5.09)	5.89 (2.86)	34.1 (3.4)
Two-phase	30.55 (2.44)	74.47 (3.51)	43.23 (2.42)	17.17 (1.47)	9.63 (0.93)	33.2 (1.5)
Res-UNet	95.27 (0.54)	73.75 (0.42)	83.14 (0.28)	42.37 (0.33)	28.00 (0.26)	13.3 (0.7)
nnU-Net	97.63 (0.36)	77.81 (0.83)	86.60 (0.44)	42.97 (0.59)	28.29 (0.49)	9.8 (0.7)
MSLNet	97.43 (0.35)	85.44 (0.55)	91.04 (0.18)	45.27 (0.17)	30.06 (0.14)	8.1 (0.5)
MSLNet-Lor	96.13 (0.26)	90.70 (0.42)	93.34 (0.17)	49.20 (0.08)	33.27 (0.07)	5.8 (0.2)
95% Confidence intervals						
SCNN	9.14 26.20	74.76 89.34	15.50 39.74	5.82 15.80	3.09 8.69	30.77 37.43
Two-phase	28.16 32.94	71.03 77.91	40.86 45.60	15.73 18.61	8.72 10.54	31.73 34.67
Res-UNet	94.74 95.80	73.34 74.16	82.87 83.41	42.05 42.69	27.75 28.25	12.61 13.99
nnU-Net	97.28 97.98	77.00 78.62	86.17 87.03	42.39 43.55	27.81 28.77	9.11 10.49
MSLNet	97.09 97.77	84.90 85.98	90.86 91.22	45.10 45.44	29.92 30.20	7.61 8.59
MSLNet-Lor	95.88 96.38	90.29 91.11	93.17 93.51	49.12 49.28	33.20 33.34	5.60 6.00
Trained on CathAction, tested on Guidewire						
Res-UNet	77.16 (1.66)	24.31 (0.93)	36.96 (1.23)	18.23 (0.54)	10.45 (0.32)	43.9 (2.1)
nnU-Net	84.80 (4.07)	28.88 (4.94)	42.88 (5.72)	19.90 (2.00)	11.31 (1.19)	47.4 (6.8)
MSLNet	79.85 (2.50)	35.41 (3.82)	49.00 (4.05)	22.01 (1.31)	12.91 (0.81)	38.8 (3.2)
MSLNet-Lor	72.47 (2.63)	39.71 (6.56)	51.12 (5.96)	18.64 (1.38)	10.65 (0.80)	31.4 (4.1)
95% Confidence intervals						
Res-UNet	75.53 78.79	23.40 25.22	35.75 38.17	17.70 18.76	10.14 10.76	41.84 45.96
nnU-Net	80.81 88.79	24.04 33.72	37.27 48.49	17.94 21.86	10.14 12.48	40.74 54.06
MSLNet	77.40 82.30	31.67 39.15	45.03 52.97	20.73 23.29	12.12 13.70	35.66 41.94
MSLNet-Lor	69.89 75.05	33.28 46.14	45.28 56.96	17.29 19.99	9.87 11.43	27.38 35.42

**Table A4:** Localization results evaluated at the sequence level. Shown are the mean (and std) obtained on the guidewire test set from four independent runs.

Method	Precision	Recall	$F_{1}$	Dice	IOU	AHD
Res-UNet	95.85 (1.21)	63.55 (0.70)	76.42 (0.28)	35.79 (0.22)	22.85 (0.19)	28.3 (0.3)
MSLNet	96.87 (0.33)	85.53 (0.59)	90.84 (0.23)	38.16 (0.12)	24.31 (0.09)	8.4 (0.6)
MSLNet-Lor	95.84 (0.24)	90.26 (0.43)	92.97 (0.19)	37.73 (0.06)	23.98 (0.06)	6.2 (0.2)
95% Confidence intervals						
Res-UNet	94.66 97.04	62.86 64.24	76.15 76.69	35.57 36.01	22.66 23.04	28.01 28.59
MSLNet	96.55 97.19	84.95 86.11	90.61 91.07	38.04 38.28	24.22 24.40	7.81 8.99
MSLNet-Lor	95.60 96.08	89.84 90.68	92.78 93.16	37.67 37.79	23.92 24.04	6.00 6.40

## Appendix C

Example segmentation results on the guidewire and CathAction test sets are shown in Figure A1 and Figure A2, respectively.

Example localization results on the guidewire and CathAction test sets are shown in Figure A3 and Figure A4, respectively.

In Figure A5 are shown results on the guidewire dataset. In Figure A5b are shown are results of the proposed segmentation architecture with the MSL branch are shown, while in Figure A5c,d are shown the MSLNet-Lor segmentation and localization results, respectively. From Figure A5, one could see that the MSL branch improves the segmentation accuracy while the localization thins the segmentation result and fills some gaps based on good continuation.
